# Monkeypox virus genome sequence from an imported human case in Colombia

**DOI:** 10.7705/biomedica.6647

**Published:** 2022-09-02

**Authors:** Katherine Laiton-Donato, Diego A. Álvarez-Díaz, Carlos Franco-Muñoz, Héctor A. Ruiz-Moreno, Paola Rojas-Estévez, Andrés Prada, Alicia Rosales, Martha Lucía Ospina, Marcela Mercado-Reyes

**Affiliations:** 1 Grupo Genómica de Microorganismos Emergentes, Dirección de Investigación en Salud Pública, Instituto Nacional de Salud, Bogotá, D.C., Colombia Universidad Nacional de Colombia Instituto Nacional de Salud Bogotá, D.C. Colombia; 2 Dirección General, Instituto Nacional de Salud, Bogotá, D.C., Colombia Universidad Nacional de Colombia Instituto Nacional de Salud Bogotá, D.C. Colombia

**Keywords:** Monkeypox virus, nanopore sequencing, phylogeny, Colombia, virus de la viruela de los monos, secuenciación de nanoporos, filogenia, Colombia

## Abstract

**Introduction:**

*Monkeypox virus* (MPXV) is an enveloped double-stranded DNA virus with a genome of approximately 197.209 bp. The current classification divides MPXV into three clades: Clade I (Central African or Congo Basin clade) and clades IIa and IIb (West African clades).

**Objective:**

To report the complete genome and phylogenetic analysis of a human monkeypox case detected in Colombia.

**Materials and methods:**

Exudate from vesicular lesions was obtained from a male patient with recent travel history to Spain. A direct genomic approach was implemented in which total DNA from the sample was purified through a column-based method, followed by sequencing on the Nanopore GridION. Reads were aligned against the MPXV reference genome using minimap2 v.2.24 and phylogenetic inference was performed using maximum likelihood estimation.

**Results:**

A total of 11.951 reads mapped directly to a reference genome with 96.8% of coverage (190.898 bp).

**Conclusion:**

Phylogenetic analysis of the MPXV circulating in Colombia demonstrated its close relationship to clade IIb responsible for the multi-country outbreak in 2022.

## Resumen

**Introducción.:**

El virus de la viruela del mono (MPXV) está compuesto por un genoma de ADN bicatenario, aproximadamente, de 197.209 pb. La clasificación actual agrupa el MPXV en tres clados: clado I (de la cuenca del Congo en África central), y clados IIa y IIb (de África occidental).

**Objetivo.:**

Reportar el genoma completo y el análisis filogenético de un caso humano de viruela símica detectado en Colombia.

**Materiales y métodos.:**

Se obtuvo exudado de lesiones vesiculares de un paciente varón con el antecedente de un viaje reciente a España. Se implementó un enfoque directo, en el cual se purificó el ADN total de la muestra mediante un método basado en columnas, seguido de la secuenciación directa en la plataforma Nanopore GridION. Las lecturas se alinearon con el genoma de referencia del MPXV, utilizando minimap2, v.2.24, y la inferencia filogenética fue realizada mediante la estimación por máxima verosimilitud.

**Resultados.:**

Un total de 11.951 lecturas se alinearon directamente con el genoma de referencia con una cobertura del 96,8 % (190.898 pb).

**Conclusión.:**

El análisis filogenético del MPXV circulante en Colombia demostró su estrecha relación con el clado de África occidental (clado IIb) responsable del brote en múltiples países en el 2022.

Monkeypox virus (MPXV) is a zoonotic pathogen associated with a febrile rash disease in humans. It has caused multiple outbreaks in the Africa ^1^ and since May 13, 2022, human cases of monkeypox were identified in 12 nonendemic African countries in Europe, Australia and North America. Individuals were infected with the West African clade and cases were mainly but not exclusively reported amongst men who have sex with men (MSM) ^2^.

*Monkeypox virus* (MPXV) is composed of a double-stranded DNA genome of approximately 197.209 bp. Two genetic clades have been characterized: West African and Central African. However, a new classification has been implemented by the WHO: clades I, IIa, and IIb ^3^^,^^4^. The current international 2022 clade is named B.1. On July 1^st^., 2022, 5,783 cases were reported in 52 countries ^5^. In Colombia there were 5 imported cases from Europe until July 5. Here we report the complete genome and phylogenetic analysis of a human monkeypox case detected in Colombia.

## Materials and methods

### Direct sequencing

Exudate from vesicular lesions was received on June 23, 2022, from a male patient with recent travel history to Spain. This was a complex sample that contained genetic material from the host and microbiome, and other coinfections.

Total DNA purification was performed using 200 μl of sample and the PureLink Viral RNA/DNA Mini Kit™ (Life Technologies, USA), according to the manufacturer’s instructions. DNA was quantified by fluorimetry with the Qubit dsDNA High Sensitivity Assay™ (Life Technologies, USA) on the Qubit 4.0™ instrument (Life Technologies, USA). Sequencing was performed using 400 ng of DNA using the native barcode kit EXP-NBD196™ (Oxford Nanopore Technologies ONT, UK) and a 1:1 ratio of AMPure XP™ beads (Beckman Coulter, UK). The library was loaded onto FLO-MIN106™ flow cells on the GridION™ sequencer (Oxford Nanopore Technologies ONT, UK).

Basecalling and demultiplexing were performed on nanopore sequence reads using Guppy™, v.6.1.7 (Oxford Nanopore Technologies), and adapters were trimmed by using Porechop™, version 0.2.4. Processed reads were aligned against the MPXV reference genome (GenBank reference No. NC063383.1) using minimap2, v.2.24 ^6^. Variant calling for single-nucleotide variants was performed with Medaka, v.1.15.0. Sites with depth less than 10x were masked with Ns. maximum likelihood phylogenetic reconstruction was performed on the alignment with 22 genomes using IQ-TREE software ^7^, K3Pu+F+l nucleotide substitution model, and bootstrap for branch support (UFBoot) with 1000 replicates. Variant calling as single nucleotide polymorphism (SNP) and multiple nucleotide polymorphism (MNP) were cross-checked by two methods through manual curation, using Snippy, v.4.6.0, and samtools pileup, v.1.15 ^8^.

### Ethics

According to the national law 9/1979, decrees 786/1990 and 2323/2006, the *Instituto Nacional de Salud* is the reference lab and health authority of the national network of laboratories and in cases of public health emergency or those in which scientific research for public health purposes as required, the *Instituto Nacional de Salud* may use the biological material for research purposes, without informed consent, which includes the anonymous disclosure of results.

This study was performed following the ethical standards of the Declaration of Helsinki 1964 and its later amendments. The information used in this study comes from secondary sources of data that were previously anonymized and do not represent a risk to the community.

## Results

A total of 11.951 reads mapped directly to a reference genome with 96.8% of coverage (190.898 bp), and the consensus sequence was submitted to the GISAID database. The sequence is available under the GISAID accession ID EPI_ISL_13511312.

Analysis by BLASTn shows a 98.77% identity to MPXV Clade l (Accession NC003310.1) and 99.42% identity to MPXV Clade IIb (Accession ON568298.1).

**Figure 1:**
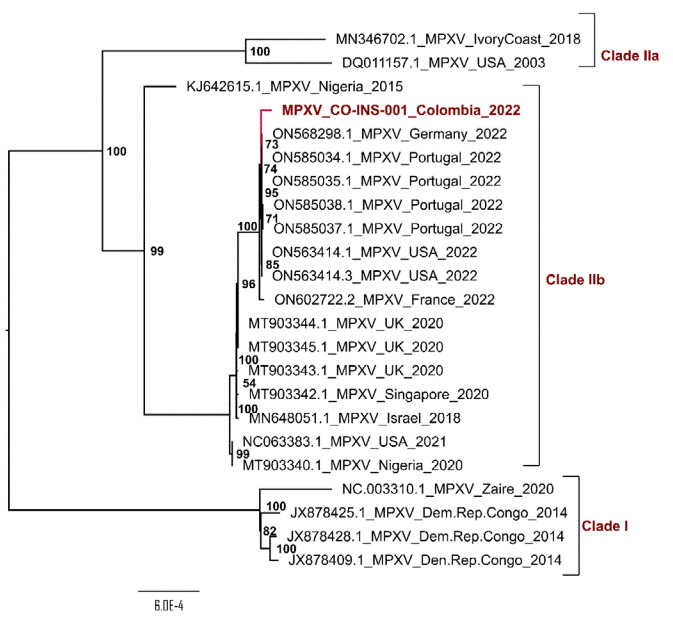
Phylogenetic tree of Monkeypox virus. The tree was reconstructed by maximum likelihood with the estimated K3Pu+F+I nucleotide substitution model for the dataset of 23 genomes and 1000 bootstrap replicates.

Phylogenetic analysis of the MPXV genome circulating in Colombia with genome sequences from NCBI (table 1) demonstrated its close relationship to Clade IIb (previously known as West African clade) and to genomes described during the multi-country outbreak in 2022 (figure 1).

**Table 1:** MPXV genomes

Accession	Country	Year	Reference
JX878425	USA	2014	(Kugelman, *et al*., 2014) ^9^
JX878428	USA	2014	(Kugelman, *et al*., 2014) ^9^
JX878409	USA	2014	(Kugelman, *et al.*, 2014) ^9^
MN346702	Berlin	2018	(Patrono, *et al*., 2020) ^10^
DQ011157	USA	2003	(Likos, *et al.*, 2005) ^11^
KJ642615	Nigeria	2015	(Nakazawa, *et al*., 2015) ^12^
CO-001	Colombia	2022	This work
ON568298	Germany	2022	(Antwerpen, *et al.*, 2022) ^13^
ON585034	Portugal	2022	(Isidro, *et al*., 2022) ^14^
ON585035	Portugal	2022	(Isidro, *et al.*, 2022) ^14^
ON585038	Portugal	2022	(Isidro, *et al.*, 2022) ^14^
ON585037	Portugal	2022	(Isidro, *et al.*, 2022) ^14^
ON563414	USA	2022	(Gigante, *et al.*, 2022) ^15^
ON602722	France	2022	(Croville, *et al.*, 2022) ^16^
MT903344	UK	2018	(Mauldin, *et al.*, 2022) ^17^
MT903345	UK	2018	(Mauldin, *et al*., 2022) ^17^
MT903343	UK	2018	(Mauldin, *et al*., 2022) ^17^
MT903342	Singapore	2019	(Mauldin, *et al.*, 2022) ^17^
MN648051	Israel	2018	(Cohen-Gihon, *et al*., 2020) ^18^
NC_063383	Nigeria	2022	(Mauldin, *et al.*, 2022) ^17^
MT903340	Nigeria	2018	(Mauldin, *et al*., 2022) ^17^
NC_003310	Russia	2020	(Shchelkunov, *et al.*, 2021) ^19^

## Discussion

A complete genome sequence was successfully obtained with the described approach. This strategy allows the assembly of a full genome without viral culture and without amplification of viral DNA. The assembled preserves a close relation to samples from the 2022 MPXV outbreak. However, due to the possibility of artifacts proper of the sequencing technology used, manual curation of called variants is necessary as implemented in this work.

Microevolution of MPXV has been observed worldwide in the sequences of the 2022 outbreak ^20^^,^^21^. It is necessary to continue the genomic surveillance of MPXV in order to detect possible changes in transmission.

In Colombia, real-time genomic surveillance and the implementation of NGS sequencing methods allowed the early detection of the introduction of MPXV in the country. This strategy will be established to monitor secondary autochthonous cases to describe local viral evolution during the transmission, characterize local transmission dynamics, study the impact of imported cases, and track viral diversity.
